# Notes and News

**Published:** 1893-04-08

**Authors:** 


### Notes and News.

Collected and Communicated.

Necessary as the punishment of crime is for the well-being of the many, there can be no doubt that the effect of prison life on the individual has a lowering tendency. The moment of leaving the prison walls is one when sympathy and encouragement is most needed. Society has much for which to thank that excellent mission, the London Police Court Mission, which endeavours to aid and restore self-respect to a large number of the population who, without assistance at the right moment, would help to swell the pauper and criminal classes of the country. Last year the mission found situations for 1,500 men, women, boys, and girls. The association has a labour yard at Ealing, and here many men are initially employed, who afterwards are provided with situations and become useful members of society. This rescue work is not accomplished without a large outlay, and the efforts of the society should not be hampered by need of the necessary funds.

Public attention has been lately much drawn to the subject of the proper ventilation of school buildings in England, and it is felt that the health of children can be seriously impaired by daily inhaling foul air. It is admitted that an undue proportion of carbonic acid gas in the air is most injurious to health, yet until quite recently no efforts have been made to ascertain whether this undue proportion does or does not exist in the atmosphere of schools in which so large a portion of the youth of the population is past. In America reform is felt to be very necessary also. In several States great improvement has been effected, and now in New York the matter has been taken up very seriously. The Boards of Health and of Education have instituted a thorough investigation of the subject throughout the country. The result has been that it is decided to establish a standard of air supply for the New York schools of 30 cubic feet of air per minute to each pupil. The method to attain this is not regulated. One important building has adopted force air currents, air tempering before heating, and the use of the Johnston heat regulating device. The air movement is induced by an Otto gas engine. During cold weather the air will be drawn from the outside to the fan, passing over heating stacks, and through built-in flues to the rooms. The foul air is driven off through flues extending from the flooring to above the roof.

The annual public meeting of the Church of England Society for Providing Homes for Waifs and Strays will be held on Monday, May 15th, at three p.m., the Earl of Leven and Melville in the chair.

The International Medical Congress for 1893, which will take place in Rome in September next, is to consist of eighteen sections. Dr. Baccelli is to be President; five volumes of Transactions will be published, to cost £1, and they will form a most valuable addition to medical libraries.

Medical men in New Zealand are complaining bitterly of the unrestricted practices of the druggists. One doctor gives instances of the grossest malpractices of a chemist, who, besides prescribing, attended medical and surgical cases, the doctor and the victims alike having no power of appeal. It is suggested that the medical profession in New Zealand should affiliate with the London Medical Protection Society.

In connection with the Owens College Hospital, Manchester, a Cancer Pavilion and Home has been established. Later on it is proposed to have a model hospital for the reception of patients suffering from cancer; but, in the meanwhile, the Owens College Hospital have acquired Stanley House and grounds, and here patients are now admitted, either as incurables or as subjects for treatment with a view to cure. All will be done that is possible to minimise the sufferings attendant on this terrible disease, and still more will be feasible when the new building is erected, towards which subscriptions are now asked.

Statistics from New Zealand, which show that male patients suffering from cancer exceed the number of females, have been the subject of much discussion. Several reasons are given as an explanation of facts so contrary to the experience of other countries. In New Zealand the male population exceeds the female, and food and habits are such as to induce the disorder. The diet in districts far from the centres of civilisation consists, with very little variation, of tea, a species of griddle cake, and mutton—a diet little varied and badly cooked, and conducive to indigestion.

Now that reform is being applied to the sanitary condition of schools in America, it is not surprising that mental hygiene should also be a subject of inquiry and proposed amelioration. Undoubtedly youth is the time for learning, but being the time for growth also anything that retards natural expansion acts adversely on the value of the knowledge to be acquired. It has been observed that "habitual truants exhibit superior physical stamina to that of the average of pupils regularly attending school, and undoubtedly the most physically capable have the advantage in the long run. Commence with the kindergarten system, and proceeding to more advanced grades of education with hours of work more varied, no home lessons allowed, and properly supervised playgrounds, and school will be no longer irksome, and healthier bodies and minds will result. Even in these days of competition and endless examinations, we venture to predict better results for the pupil brought up under hygienic principles applied mentally and bodily.

